# Factors contributing to road crashes among commercial vehicle drivers in the Kintampo North Municipality, Ghana in 2017

**DOI:** 10.4314/gmj.v54i3.2

**Published:** 2020-09

**Authors:** Samuel A Poku, Delia A Bandoh, Ernest Kenu, Emma E Kploanyi, Adolphina A Addo-Lartey

**Affiliations:** 1 College of Health and Well-Being, Kintampo, Bono East Region, Ghana; 2 School of Public Health, University of Ghana, Legon, Accra, Ghana

**Keywords:** commercial drivers, road crashes, vehicle, road signs, traffic light signal

## Abstract

**Objective:**

The study assessed driver, vehicular and road-related factors associated with road crashes (RC) in the Kintampo North Municipality.

**Design:**

Cross-sectional study

**Setting:**

Kintampo North Municipality

**Data source:**

Demographics, vehicular and road usage information on registered drivers at Ghana Private Road and Transport Union (GPRTU) and Progressive Transport Owners Association (PROTOA) in Kintampo North Municipality

**Main outcome:**

involvement in road crashes and related factors

**Result:**

A total of 227 drivers were approached for this study. None of them declined participation. They were all males. Most were between 28–37 years (30%). The proportion of drivers that reported RC ever involvement in at least one RC was 55.5% (95% CI: 8.0%, 62.1%). In the bivariate analysis, drink and drive changed lane without signalling, ever bribed police officer, drove beyond the maximum speed limit, paid a bribe at DVLA for driving license, violation of traffic signals were found to be associated with RC involvement (p<0.05). Drivers who violated traffic signals had 2.84 odds of being involved in road crashes compared to those who did not [aOR; 2.84 (95%CI:1.06,7.63)]

**Conclusion:**

The proportion of drivers ever involved in road crashes was high. The major factor that is associated with RC involvement was a violation of the traffic light signals. Continuous driver education and enforcement of road traffic regulations by the appropriate authorities could curb the road crash menace in the Municipality.

**Funding:**

The authors funded this work.

## Introduction

Globally more than 1.2 million people lose their lives yearly as a result of road crashes^1^. The United States Centres for Disease Control has also reported that each day, an estimated 3,400 people are killed globally in road crash^2^. The evidence further shows that 70% of the world's road fatalities occur in developing countries 3. Data compiled by the Motor Traffic and Transport Department (MTTD) in Ghana revealed that in 2017, 12,843 road crashes occurred out of which 2,076 people died and 12,166 were injured^4^. Similarly, the Brong Ahafo Region recorded a total of 503 road crash, 190 deaths, and 313 injured persons in 2017. The Kintampo North Municipality, recorded 79 crashes, 30 deaths, and 255 injured persons in the year 2017.

Commercial vehicles were also more involved in road crashes compared to private vehicles in the Kintampo North Municipality^5^.

According to the World Health Organization, the adoption and implementation of respectable laws by countries on the main predisposing factors associated with road traffic injuries essentially change the behaviour of road users^1^. The five “Es” strategy: Enforcement, Education, Evaluation, Engineering, and Encouragement were emphasised by Adamos et al. as a strategy to reduce road crash^6^. Currently, interventions to reduce road crashes in Ghana include traffic calming, road bypass of major settlements, identification and treatment of black spots, and law enforcement against drunk-driving^7^.

The Kintampo North Municipality regularly embarks on road safety campaigns and enforcement of the road traffic regulations to reduce road crash. However, people continue to die and sustain injuries through a road crash. A report by the National Road Safety Commission revealed the need for in-depth research to provide a better understanding of the current road safety challenges in Ghana^7^. This research was therefore carried out to identify the factors contributing to the road crashes in the Kintampo North Municipality. Specifically, the study assessed the association between road crashes and the driver, vehicular and road-related factors in the Kintampo North Municipality.

## Methods

### Study design

A cross-sectional study was conducted to assess the factors contributing to road crashes among commercial vehicle drivers in the Kintampo North Municipality of Ghana from January to December 2017. The road factors, driver factors, and vehicular factors related to road crashes were obtained by asking the drivers to indicate what they have observed throughout their driving career.

### Study setting

The study was carried out in the Kintampo North Municipality in the Bono East Region of Ghana. The Municipality shares boundaries with five districts in the country namely: Central Gonja District to the North, Bole District to the West, East Gonja District to the North-East, Kintampo South District to the South, and Pru District to the South- East. The estimated population of the Municipality was 114,481 in the year 2018. The Municipality serves as a transit point between the Southern and Northern sectors of Ghana. The main means of transport in the Municipality is by road. The principal road network passing through the Municipality is the Techiman-Tamale road which is tarred and used daily by passengers primarily due to the markets in Kintampo and Techiman townships. The Kintampo-Nkoranza road, Kintampo-Newlongoro, and the other roads linking up to the Municipality are not tarred. The untarred roads in the Municipality are regularly used by farmers to transport farm produce to the Municipal market. It is difficult to find means of transport from the surrounding remote communities to the Municipality, especially during raining seasons due to the muddy nature of the untarred roads. The Municipality sees many visitors owing to its many tourist attraction sites. People from all walks of life travel to the Municipality, especially during festivities to see places such as Kintampo Water Falls, Kintampo Fuller falls, and the Monkey Sanctuary at Boabeng-Fiema community.

The Municipality has only one government Hospital, 20 government health centers, and four private health centers providing health care services to clients. The Kintampo Municipal Hospital is the main referral point for road crashes victims. Also, there is a Divisional Police Headquarters in the Municipality located at Kintampo maintaining law and order.

### Study population

The study population was Driver and Vehicle Licencing Authority (DVLA) licensed commercial vehicle drivers operating with GPRTU and PROTOA in the Kintampo North Municipality.

### Sample size

The sample size was computed using Cochran's formula^8^. The Cochran's formula: n=z^2^ p (1-p)/e^2^ was used for the estimation of the sample size. The prevalence of road crashes (15.9%) used in the sample size estimation was based on the findings of Adejugbagbe et al. on road crashes in Ibadan, Nigeria^9^. A total sample size of 227 was used for the study, assuming a 10% non-response rate.

### Sampling

Stratified sampling was used to enrol the participants. The five main commercial vehicle stations (Kumasi, Tamale, Apesika, Bomboi, and Suamere station) were used as strata. At each station, the Chairmen for the transport unions (GPRTU and PROTOA) provided the list of the total number of drivers and their vehicle numbers from the station register. The number of drivers selected from each station was proportionate to the total number of drivers at the station. The number of drivers for each station exceeded the sample proportion allotted to the station. Using simple random sampling, drivers were selected from each of the five stations. This was done by writing the vehicle numbers of the drivers on pieces of paper. The papers were folded and put into an opaque container. The container was vigorously shaken to ensure that the papers were mixed thoroughly. At each station, one of the Chairmen was randomly selected to pick the pieces of papers bearing a vehicle number one after the other without looking into the container until the proportion allotted to the station was reached. The drivers whose vehicle numbers were found on the pieces of papers picked irrespective of their transport union were interviewed.

### Data collection

Interviewer-administered semi-structured questionnaires were used to collect data on the demographic and job characteristics of drivers and their road crash involvement. The instrument for data collection was adapted from a road crash study carried out in Ghana^10^. The interviews were conducted by the principal investigator and four research assistants who were trained to assist with the data collection.

One-on-one interview (verbal) was carried out in an enclosed place at the various lorry stations where interviews could not easily be overheard. The questions were translated into the local dialect (Twi) by the interviewers. The opinion of drivers was obtained on driver-related and road-related factors. The data on driver-related factors were collected under three domains: professionalism of drivers, their road practices, and knowledge of the road signs. Vehicle-related factors included information on vehicle specifications for roadworthiness, and road-related factors indicating the availability and functioning of some road furniture.

### Data analysis

Stata® Statistics/Data Analysis software, (College Station, Texas 77845 USA) version 15 was used for the data analysis. The descriptive analysis was done using frequencies and percentages. The data were presented using tables and figures. Pearson's chi-square test and the Fishers' exact test were employed in assessing the factors that are associated with road crash involvement. Simple logistic regression was used to quantify the strength of the association between statistically significant variables and road crash involvement. Multiple logistic regression was used to adjust for the variables that were significantly associated with road crash involvement from the bivariate analysis. The significance level of statistical tests was set at p < 0.05.

### Ethical Consideration

Ethical approval was obtained from the Ethics Review Committee (ERC) of the Ghana Health Service. The ERC approval number is GHS-ERC: 058/12/17. Consent was sought from the drivers' unions, the vehicle operators, and MTTD before the study was conducted. The study was explained to the drivers, and all their questions explained to them. Each driver signed informed consent before the study interview was done. The confidentiality of the data was maintained throughout the study. Data was kept in locked cabinets and was only accesses by authorised persons.

## Results

### Background characteristics

A total number of 227 drivers participated in the study. They were all males, and most were between 28–37 years (30%). About one in five drivers (23.8%) had secondary or higher education and almost half (49.8%) drove taxis. The proportion of drivers that reported at least one road crash ever involvement was 55.5% (95%CI=8.0%–62.1%).

Thirty-five of them (27.8%) had sustained injuries and 21.4% had been hospitalised due to the road crashes (Table 1).

**Table 1:** Background characteristics of study participants

Characteristics	Frequency	Percentage
**Age of driver**		
**<18 years**	9	3.96
**18–27 years**	26	11.45
**28–37 years**	68	29.96
**38–47 years**	56	24.67
**48–57 years**	49	21.59
**>57 years**	19	8.37
**Highest Educational level**		
**None**	37	16.30
**Primary**	49	21.59
**JHS/Middle School**	87	38.33
**Secondary**	43	18.94
**Tertiary**	11	4.85
**Vehicle type**		
**Van**	105	46.26
**Taxi**	113	49.78
**Bus**	9	3.96
**Years of driving experience**		
**<5 years**	44	19.38
**6–10 years**	65	28.63
**11–15 years**	44	19.38
**16–20 years**	40	17.62
**>20 years**	34	14.98
**RC ever involvement**		
**No**	101	44.50
**Yes**	126	55.50
**Sustained injury in RC**		
**No**	91	72.22
**Yes**	35	27.78
**Hospitalised in RC**		
**No**	99	78.57
**Yes**	27	21.43

**Figure 1:**
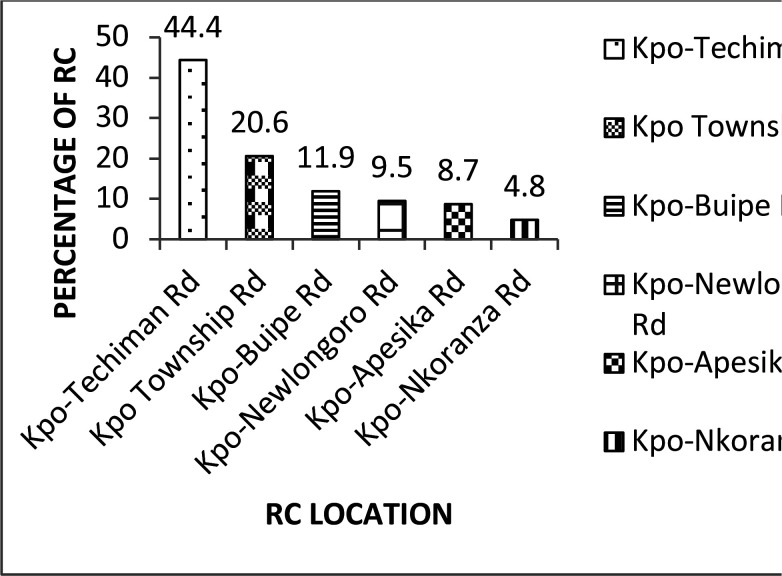
Location of road crashes in the Kintampo North Municipality

Most (44.4%) of the drivers who indicated having ever been involved in road crashes mentioned that the accidents occurred on the Kintampo-Techiman road. The Kintampo-Nkoranza road recorded the least (4.8%) accidents (Figure 1).

### Bivariate analysis of the association between road crashes involvement and background characteristics

The bivariate analysis presented in Table 2 showed that the type of vehicle operated by the drivers was significantly associated with road crashes involvement (p<0.034).

**Table 2:** Bivariate analysis of the association between background characteristics and road crashes involvement

	Involved in RC				
Characteristics	Total	No (%)	Yes (%)	χ^2^	P-value
**Age of driver**				4.13	0.531
**<18 years**	9 (3.96)	2 (1.98)	7 (5.56)		
**18–27 years**	26 (11.45)	13 (12.87)	13 (10.32)		
**28–37 years**	68 (29.96)	33 (32.67)	35 (27.78)		
**38–47 years**	56 (24.67)	21 (20.79)	35 (27.78)		
**48–57 years**	49 (21.59)	24 (23.76)	25 (19.84)		
**>57 years**	19 (8.37)	8 (7.92)	11 (8.73)		
**Highest educational level**				6.73	0.151
**None**	37 (16.30)	21 (20.79)	16 (12.70)		
**Primary**	49 (21.59)	22 (21.78)	27 (21.43)		
**JHS/Middle School**	87 (38.33)	31 (30.69)	56 (44.44)		
**Secondary**	43 (18.94)	23 (22.77)	20 (15.87)		
**Tertiary**	11 (4.85)	4 (3.96)	7 (5.56)		
**Driver union**				0.06	0.799
**GPRTU**	135 (59.47)	61 (60.40)	74 (58.73)		
**PROTOA**	92 (40.53)	40 (39.60)	52 (41.27)		
**Vehicle type**				6.76	0.034
**Van**	105 (46.26)	54 (53.47)	51 (40.48)		
**Taxi**	113 (49.78)	46 (45.54)	67 (53.17)		
**Bus**	9 (3.96)	1 (0.99)	8 (6.35)		
**Years of driving experience**				3.28	0.509
**<=5 years**	44 (19.38)	18 (17.82)	26 (20.63)		
**6–10 years**	65 (28.63)	33 (32.67)	32 (25.40)		
**11–15 years**	44 (19.38)	22 (21.78)	22 (17.46)		
**16–20 years**	40 (17.62)	16 (15.84)	24 (19.05)		
**>20 years**	34 (14.98)	12 (11.88)	22 (17.46)		

χ^2^: Pearson's chi-square value. %: column percentage. RC: Road Crash. GPRTU: Ghana Private Road Transport Union. PROTOA: Progressive Transport Owners Association.

All driver factors assessed were significantly linked with involvement in a road crash. None of the road and vehicular factors assessed was associated with road crashes involvement.

### Multivariate analysis of the factors associated with road crashes involvement

Drink and drive, changed lane without signalling, ever bribed police officer, drove beyond the maximum speed limit, and bribed at DVLA for driving license was found to be associated with road crashes involvement (p<0.05). After adjusting for confounders, drivers who violated traffic signals had 2.84 odds of being involved in road crashes compared to those who did not violate traffic signals (p<0.05).

## Discussion

This study assessed the factors contributing to road crashes in the Kintampo North Municipality. We found that the type of vehicles driven by the drivers, drunk-driving, violation of red traffic light signal, changing lane without signalling, alleged bribing Police Officers, overloading of vehicles, speeding, and alleged bribery at the DVLA for driving licenses were the factors associated with road crashes involvement from the bivariate analysis. The violation of the traffic light signal was the only variable that was significant from the multiple logistic regression analysis.

The World Bank report indicated that 90% of all licensed drivers speed at some point in their driving career^11^. Speeding was identified as a factor contributing to the road crashes menace in this study. This suggests that there is a need to enhance the education of drivers on the consequences of speeding.

Additionally, regulations on speed limits need to be properly enforced. Though the traffic police are bestowed with the power to enforce road traffic regulation, not much has been achieved considering the road crashes prevalence (55.5%) in this study. Many of the drivers in this study indicated that they pay bribes to Police Officers for breaching road traffic regulations. This is comparable to Odame's observation that traffic police in Ghana sometimes accept a bribe and renege on their duties to enforce the road traffic regulations^12^.

Nyoni also asserted that in Ghana, it is a common practice for drivers to be seen giving bribes to Police Officers at roadblocks^13^. Mervis has reported corruption in the issuing of driver's license and stated that drivers without proper training are one of the factors that contribute to road crashes in Ghana^14^. This is not different from the findings of this study. Some of the drivers self-reported paying a bribe at the DVLA office to obtain a driving license.

These findings buttress the fact that the MTTD and the DVLA need to step up in the enforcement of the road traffic regulations in the fight against road crash. In the present study, all the drivers were males, which is comparable to the findings of Gumah, 99.2% of commercial drivers were males^10^. Among the drivers studied, 3.96% self-reported that were under the age of 18 years with 77.8% of them reporting road crashes involvement. As per the Ghana road traffic act-2004(ACT 683), section 62, it is illegal to drive or obtain a driver's license if you are under 18 years of age.

We were however not able to verify if drivers were truly below 18 years since we did not validate the reported age using a birth certificate or national identity cards. The proportion of road crashes among drivers reported to be below 18 years was however the highest compared to the other age groups. If the reported ages are accurate, then our results suggest that underage driving predisposes driver and the passengers to a road crash. The DVLA may, therefore, have to do additional verification of the age of people applying for a driving license before issuing it to them.

The number of commercial vehicles especially taxis in the Kintampo North Municipality are expected to increase. This is because taxis are most patronised in the Municipality. The rise in the number of vehicles is likely to correspond with an increase in the number of road crashes in the Municipality. This assertion is in line with Yankson et al.'s observation that a rise in motorisation is the result of the sharp increase in road crashes in lower and middle-income countries^15^. Growder had also indicated that an increase in the number of motor vehicles is a major contributory factor in the rising toll of road fatalities and injuries in poor countries^16^.

From our study, driver factors were found to contribute to road crashes in Kintampo North Municipality. Hashmi, Qayyum & Rehman observed that more than 90% of road crashes occur due to human errors^17^. Mohanty & Gupta also noted that road geometric factors immensely contribute to road crash^18^. Nonetheless, the findings of this study might not be generalisable due to the following limitations. First of all, the cross-sectional study design used for the study is not strong enough to establish temporality or the true cause of a road crash.

The data collected for the study was also self-reported by the drivers based on how well the drivers recollected their experience. To reduce this bias, we assured drivers of the confidentiality of the information and tried to make them comfortable so that they could share all their experiences and knowledge.

We also probed using major events reported in the media to help them remember what had happened to them. Therefore, the associations seen in this study should be interpreted with caution.

## Conclusion

The proportion of drivers ever involved in road crashes was high. After assessing factors contributing to road crash among commercial vehicle drivers in the Kintampo North Municipality, the violation of the traffic light signal was the main factor identified. Enforcing compliance to traffic light signal can lead to the reduction in road traffic accidents in the municipality.

## Figures and Tables

**Table 3:** Bivariate analysis of the association between road crash involvement and driver, vehicular, and road factors

	Involved in RC (%)				
Characteristics	Total	No (101)	Yes (126)	χ^2^	P-value
**Driver Factor**					
**Drinks and drive**				5.91	0.015
**No**	184 (81.06)	89 (88.12)	95 (75.40)		
**Yes**	43 (18.94)	12 (11.88)	31 (24.60)	ASS	
**Violates the red traffic signal**				14.31	<0.001
**No**	190 (83.70)	95 (94.06)	95 (75.40)		
**Yes**	37 (16.30)	6 (5.94)	31 (24.60)		
**Changes lanes without signalling**				9.62	0.002
**No**	176 (77.53)	88 (87.13)	88 (69.84)		
**Yes**	51 (22.47)	13 (12.87)	38 (30.16)		
**Bribes police officers**				6.51	0.011
**No**	87 (38.33)	48 (47.52)	39 (30.95)		
**Yes**	140 (61.67)	53 (52.48)	87 (69.05)		
**Overloads vehicle**				5.04	0.025
**No**	66 (29.07)	37 (36.63)	29 (23.02)		
**Yes**	161 (70.93)	64 (63.37)	97 (76.98)		
**Speeding**				9.26	0.002
**No**	116 (51.10)	63 (62.38)	53 (42.06)		
**Yes**	111 (48.90)	38 (37.62)	73 (57.94)		
**Bribed at DVLA for driving license**				10.41	0.001
**No**	162 (71.37)	83 (82.18)	79 (62.70)		
**Yes**	65 (28.63)	18 (17.82)	47 (37.30)		
**Vehicular Factors**					
**Functioning mirror**				Ψ	0.385
**No**	5 (2.20)	1 (0.99)	4 (3.17)		
**Yes**	222 (97.80)	100 (99.01)	122 (96.83)		
**Functioning speedometer**				0.1	0.747
**No**	79 (34.80)	34 (33.66)	45 (35.71)		
**Yes**	148 (65.20)	67 (66.34)	81 (64.29)		
**Functioning seatbelts**				0	0.962
**No**	20 (8.81)	9 (8.91)	11 (8.73)		
**Yes**	207 (91.19)	92 (91.09)	115 (91.27)		
**Road Factors**					
**RC reduced by Road signs**				0.35	0.557
**No**	164 (72.25)	71 (70.30)	93 (73.81)		
**Yes**	63 (27.75)	30 (29.7)	33 (26.19)		
**RC reduced by road marks**				0.05	0.825
**No**	176 (77.53)	79 (78.22)	97 (76.98)		
**Yes**	51 (22.47)	22 (21.78)	29 (23.02)		
**RC reduced by street light**				1.75	0.186
**No**	156 (68.72)	74 (73.27)	82 (65.08)		
**Yes**	71 (31.28)	27 (26.73)	44 (34.92)		

χ^2^: Pearson's chi-square value. Ψ: Fishers' exact test. %: column percentage. RC: Road Crash.

**Table 4:** Multivariate analysis of driver factors associated with road crash involvement

Characteristics	Y	N	cOR (95% CI)	aOR (95% CI)
**Vehicle driven**				
**Bus**	8	1	Ref	Ref
**Van**	51	54	1.54 (0.9, 2.64)	1.24(0.69, 2.23)
**Taxi**	67	46	8.47 (1.02, 70.13)	8.17(0.89,74.62)
**Drink and drive**				
**No**	12	89	Ref	Ref
**Yes**	31	95	2.42 (1.17, 5.00)*	1.57(0.71,3.48)
**Violated traffic signal**				
**No**	95	95	Ref	Ref
**Yes**	31	6	5.17(2.06,12.96)**	2.84(1.06,7.63)*
**Changed lane without signalling**				
**No**	13	88	Ref	Ref
**Yes**	38	88	2.92 (1.46, 5.86)*	1.98(0.91,4.32)
**Ever bribed a police officer**				
**No**	39	48	Ref	Ref
**Yes**	87	87	2.02 (1.17, 3.48)*	1.54(0.84,2.82)
**Overloaded passenger**				
**No**	64	37	Ref	Ref
**Yes**	97	29	1.93 (1.08, 3.45)	1.39(0.73,2.67)
**Drove beyond the maximum speed limit**				
**No**	53	63	Ref	Ref
**Yes**	73	38	2.28 (1.34, 3.9)*	1.56(0.86,2.84)
**Bribed at DVLA for driving license**				
**No**	79	83	Ref	Ref
**Yes**	47	18	2.74 (1.47, 5.12)*	1.73(0.87,3.43)

cOR: Crude Odds Ratio; aOR: Adjusted Odds Ratio; CI: Confidence Interval

* p<0.05

** p<0.001
